# Evidências materiais e imateriais do território de Manguinhos

**DOI:** 10.1590/S0104-59702024000100040

**Published:** 2024-10-11

**Authors:** Renato Kipnis, Ilza Carla Favaro de Lima

**Affiliations:** i Diretor, Scientia Consultoria Científica. São Paulo – SP – Brasil rkipnis@scientiaconsultoria.com.br; ii Doutoranda, Programa de Pós-graduação em Museologia e Patrimônio/Universidade Federal do Rio de Janeiro. Rio de Janeiro – RJ – Brasil; iii Surpevisora, Scientia Consultoria Científica. São Paulo – SP – Brasil ilza.lima@scientiaconsultoria.com.br

**Keywords:** Território, Patrimônio arqueológico, Patrimônio imaterial, Licenciamento ambiental, Territory, Archaeological heritage, Intangible heritage, Environmental licensing

## Abstract

O artigo aborda o licenciamento ambiental como instrumento de identificação e preservação do patrimônio cultural. Ao longo de 2021 foram conduzidos estudos na área do campus de Manguinhos da Fundação Oswaldo Cruz e vizinhança, associados à instalação de uma rede de coleta e tratamento de esgoto pelo poder público. Os estudos evidenciaram o processo de ocupação do território e permitiram identificar vestígios de cultura material em contexto arqueológico e os bens acautelados pelo Instituto do Patrimônio Histórico e Artístico Nacional – baianas de acarajé, capoeira e literatura de cordel –, saberes e formas de expressão que testemunham a construção do território de Manguinhos, processo em constante movimento.

## Abstract (en)

This article discusses environmental licensing as an instrument for the identification and preservation of cultural heritage. Throughout 2021, studies were undertaken in and around Fundação Oswaldo Cruz’s Manguinhos campus, in Rio de Janeiro, in preparation for the introduction of a sewage system by the local authority. The studies revealed how the territory had been occupied and enabled the identification of vestiges of material culture in an archaeological context and the heritage listed by Iphan, the Brazilian federal heritage protection agency – baianas de acarajé, capoeira, and cordel literature – the knowledge, and the forms of expression that bear witness to the development of the Manguinhos territory, a process in continuous motion.

Este artigo apresenta o resultado de pesquisa, realizada entre novembro de 2020 e agosto de 2021, sobre o patrimônio material e imaterial identificado no território do *campus* de Manguinhos da Fundação Oswaldo Cruz (CMF) e vizinhança. A pesquisa foi desenvolvida no âmbito do licenciamento ambiental associado à instalação de uma rede de coleta de esgoto e de tratamento (Caldarelli, Lima, Medeiros, fev. 2021; Kipnis, set. 2020; Kipnis, Costa, dez. 2021).^1^ Foram identificados na pesquisa realizada vestígios de cultura material e abundante gama de ofícios, saberes e formas de expressão que testemunham a construção do território de Manguinhos, processo esse em constante movimento.

Sítios arqueológicos e históricos tiveram sua proteção legal pelo Patrimônio Histórico e Artísitico Nacional instrumentalizada a partir do decreto-lei n.25, de novembro de 1937, que instituiu seu principal instrumento de reconhecimento e proteção do patrimônio cultural material: o tombamento. Desde então, ao longo dos últimos 86 anos, uma série de medidas legais foi e tem sido implementada, revisada e atualizada visando identificar, reconhecer, preservar, valorizar e divulgar o patrimônio cultural nacional, seja ele material ou imaterial. Esse é um processo que ao longo do tempo tem sido, e deve ser cada vez mais, socialmente inclusivo, uma vez que os cidadãos e as cidadãs são detentores desse patrimônio.

A partir da década de 1960, junto com o aumento das preocupações ambientais, principalmente na Europa e na América do Norte, houve o reconhecimento de que não somente os recursos naturais estavam em risco, mas os recursos culturais também estavam à mercê do desenvolvimento industrial mundial. Esses recursos finitos, assim como os naturais, necessitavam, portanto, de cuidadoso gerenciamento. Logo houve por parte de vários agentes preocupados com a preservação dos recursos culturais (pesquisadores, legisladores, instituições culturais etc.) a conscientização de que os recursos materiais estavam em risco e desaparecendo rapidamente, enquanto somente uma fração da informação podia ser registrada por pesquisas e estudos. Sua sobrevivência necessitava de uma perspectiva diferente, que demandava comunicação com o mundo exterior, influenciando os processos políticos e socioeconômicos de tomada de decisão, e que deveria incluir recrutamento do grande público e fortalecimento dos instrumentos legais de proteção do patrimônio cultural.

Os debates levaram à compreensão da necessidade de realização de estudos prévios à execução de obras de impacto (Unesco, 1968), para proteção de sítios e monumentos, que passaram a ser chamados de patrimônio cultural, categoria que foi sendo atualizada com o decorrer dos anos. No Brasil, a lei federal n.3.924, promulgada em 26 de julho de 1961, é a pedra angular da proteção do patrimônio arqueológico; estabelece que tais bens, identificados ou não, são propriedade da União e, assim sendo, estão sob a proteção e guarda do poder público.

Somente a partir do início da década de 1980, entretanto, com a criação do Conselho Nacional do Meio Ambiente (Conama) e promulgação de sua resolução n.1, de 23 de janeiro de 1986, têm início os esforços de regulamentar e garantir a execução de estudos de impactos ao meio ambiente – nele incluído o patrimônio cultural – gerados por empreendimentos. Em uma retrospectiva desse processo de conscientização e obrigatoriedade de pesquisas prévias, Claudia Leal (2020, p.4) destaca a importância do conteúdo da chamada Conama 86, que incluiu o meio socioeconômico junto à “definição do ambiente que deveria ser avaliado no contexto do licenciamento ambiental – o que seria reforçado pela amplitude da noção de patrimônio da Constituição Federal de 1988”.

O primeiro ato administrativo especificamente voltado para a proteção do patrimônio cultural diante da expansão econômica, nesse caso, os bens arqueológicos, só foi sancionado em 17 de dezembro de 2002, pelo Instituto do Patrimônio Histórico e Artístico Nacional (Iphan): a portaria Iphan n.230, a qual estabelece os procedimentos para estudos visando à salvaguarda do patrimônio arqueológico com relação à instalação de empreendimentos com potencial impactante. Em 2015, essa portaria é revogada, e em seu lugar é publicada a instrução normativa Iphan n.1, de 25 de março de 2015, mais abrangente e inclusiva, a qual, em seu artigo primeiro, “estabelece procedimentos administrativos a serem observados pelo Instituto do Patrimônio Histórico e Artístico Nacional (Iphan), quando instado a se manifestar nos processos de licenciamento ambiental federal, estadual e municipal em razão” de empreendimentos com potencial de impactos negativos em bens culturais acautelados em âmbito federal.

Importante salientar que os bens acautelados a que a instrução normativa se refere formam o conjunto de bens com proteção legal federal, instaurado por normas que propiciam, por exemplo, o “tombamento”, para bens materiais, por meio do decreto-lei n.25/1937, o “registro”, para bens imateriais, conforme o decreto n.3.551/2000, e a proteção dos bens arqueológicos, pela lei federal n.3.924. Na composição deste último grupo incluem-se sítios arqueológicos que ainda não são conhecidos (p. ex. enterrados) ou não listados no Cadastro Nacional de Sítios Arqueológicos do Iphan. O que o diferencia dos demais bens, pois, pela lei de tombamento, são considerados apenas os bens propostos e aceitos para inscrição em um dos quatro Livros do Tombo (Arqueológico, Etnográfico e Paisagístico; Histórico; Belas Artes e das Artes Aplicadas); no caso dos patrimônios imateriais, o decreto institui a proteção a partir da proposta e o aceite para registro em outros quatro Livros de Registro (Saberes; Celebrações; Formas de Expressão e Lugares).

As pesquisas realizadas no âmbito do licenciamento ambiental tratado neste artigo permitiram levantar informações sobre a presença de patrimônio material e imaterial na área de influência dos empreendimentos no território^2^ de Manguinhos, viabilizando a prevenção e, em casos particulares, a mitigação ou compressão de impactos negativos. Os estudos possibilitam a evidenciação dos bens, sua proteção e a informação da comunidade próxima, a maior interessada na atualização da história e da memória, e na valorização da cultura local. Também contribuem com os registros científicos, análises e propostas de ações mais conectadas com o território.

Vestígios do passado constantemente acabam soterrados por “camadas de sedimentos e de concreto” ao longo do tempo e das transformações contínuas das cidades (Andrade et al., 2020, p.8). Intervenções urbanas, como a obra em questão neste estudo, quando comprometidas com os procedimentos previstos pelas leis do licenciamento ambiental, oportunizam o encontro com tais elementos esquecidos ou escondidos. O mesmo ocorre com as referências culturais do presente que, mesmo tendo valor para os detentores e o reconhecimento dos grupos envolvidos, podem passar despercebidas em meio aos diversos movimentos de reorganização urbana.

O potencial do sítio arqueológico de Manguinhos, que gradualmente tem sido revelado nas pesquisas realizadas no *campus*, tem possibilitado, por meio do tratamento adequado dos vestígios, melhores visualização e compreensão dos processos de ocupação do território. As ações de divulgação científica e comunicação museológica, aliadas ao circuito fomentado pelos educadores da instituição, colaboram com uma visão espacial, histórica e territorial mais integrada, com a construção desses conhecimentos (Andrade et al., 2020). E as manifestações culturais contemporâneas se somam a esse conjunto múltiplo. Os bens culturais das comunidades se entrecruzam com a presença do sítio arqueológico de Manguinhos e formam a atual e desafiadora ocupação do território que, quanto mais articulada, mais pode se fortalecer.

“Não é mais possível imaginar a preservação sem o cidadão. Sem o detentor. Sem o proprietário... Sem a necessária legitimação social dos atos que, por atribuição legal, nos cabem executar” (Iphan, s.d., p.3). A população em geral e as instituições, caminhando juntas, podem participar do reconhecimento e da proteção dos seus bens culturais. É importante destacar que os estudos realizados no contexto apresentado seguem a regulamentação legal, ou seja, são estritos aos bens acautelados no nível federal. Nesse sentido, certas referências culturais que podem ser consideradas inegavelmente importantes para as comunidades, mas que ainda não passaram pelo percurso da patrimonialização institucionalizada, não são contempladas nessas análises. Essa questão reafirma a necessidade das ações articuladas de divulgação e projetos educativos que oportunizem, aos diversos grupos formadores da sociedade brasileira, a reflexão sobre sua cultura e a apreensão dessas ferramentas de visibilidade e proteção do patrimônio cultural no cenário das políticas ambientais e culturais.

## Coletor Tronco Faria-Timbó da Estação de Tratamento de Esgoto Alegria

**Figura 1:**
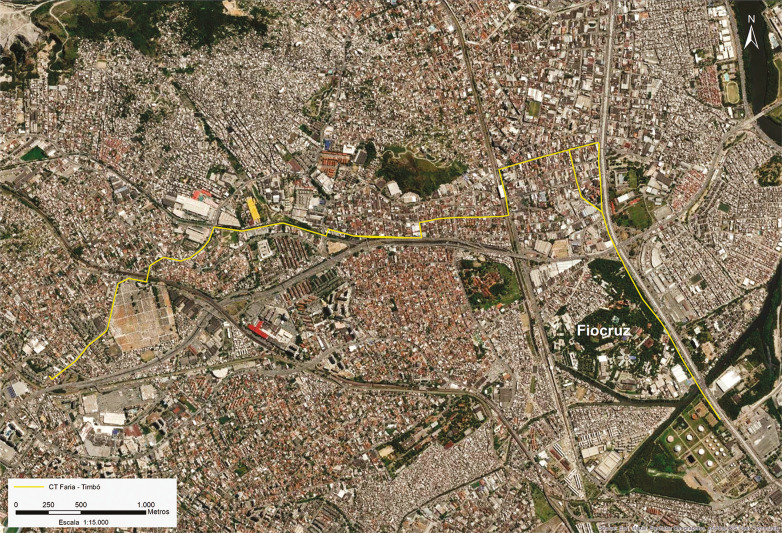
: Localização do empreendimento (GoogleEarth/Passarelli, autoria Renato Kipnis)

O Coletor Tronco (CT) Faria-Timbó é uma rede de coleta de esgoto e de tratamento na Estação de Tratamento de Esgoto Alegria, com cerca de 6km de extensão, passando pelos bairros de Ramos, Bonsucesso, Olaria, Del Castilho, Inhaúma, Tomás Coelho, Engenho da Rainha, entre outros, na cidade do Rio de Janeiro (Figura 1). Sob a coordenação da Secretaria de Estado do Ambiente e Sustentabilidade, a Construtora Passarelli foi contratada para execução da obra que previa intervenções pontuais com escavações de poços de acesso circulares, de diâmetro variando entre 5,0 e 7,5m, com profundidade próxima de 10m e, com um equipamento específico, realização de túneis na profundidade estimada de 10m, instalando simultaneamente os tubos coletores. Na superfície e subsuperfície rasa, matriz com potencial de impacto aos bens de cultura material, são, portanto, apenas as escavações de poços, em distâncias entre 100 e 300m. Características essas que, aliadas à ausência de sítios arqueológicos conhecidos no percurso do empreendimento, segundo a instrução normativa Iphan n.1, de 25 março de 2015, apresentam baixíssimo potencial arqueológico, necessitando apenas do compromisso do empreendedor de que, no caso de identificação de bens arqueológicos, seja parada a obra no local e o órgão responsável (Iphan) seja notificado.

**Figura 2:**
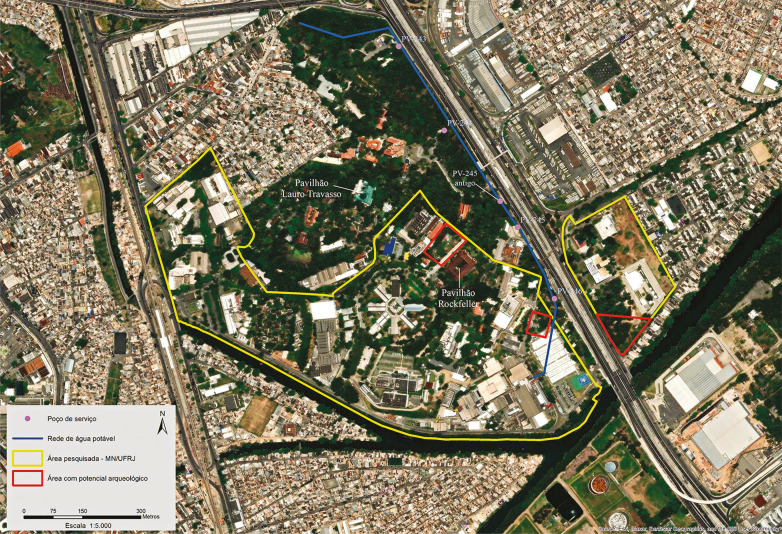
: Poços de serviço do Tronco Coletor Faria-Timbó-Fiocruz e áreas que foram objeto de pesquisas arqueológicas (GoogleEarth/Passarelli, autoria Renato Kipnis)

No entanto, pelos fatos de o traço do CT Faria-Timbó atravessar o *campus* de Manguinhos e de este ser área de interesse arqueológico, o Iphan indicou a necessidade de acompanhamento arqueológico para a obra nesse trecho. Para a implantação do CT Faria-Timbó que atravessa o CMF estava programada a abertura de quatro poços de serviço circulares, chamados de poços de visita (PV), e a instalação de tapumes provisórios no entorno de cada poço (Figura 2).

No caso específico desse trabalho, todas as atividades de escavação e movimentação de solo foram acompanhadas por uma arqueóloga. As intervenções consistiram, basicamente, em atividades de abertura de sondagens, abertura de trincheiras e abertura do PV propriamente dito, por meio de escavações manuais e com auxílio de escavadeira hidráulica.

O acompanhamento arqueológico é entendido como um tipo de intervenção arqueológica específico (Silva, 2005), muito empregado em vários países (Cifa, 2014; Smit, Van Heeringen, Theunissen, 2006; Willems, Brandt, 2004), às vezes como alternativa à arqueologia preventiva, que comumente se emprega no Brasil, com forte influência dos países europeus. Muito comuns nas áreas densamente urbanizadas, a par de ações preventivas onde elas são possíveis, os procedimentos (arqueologia preventiva e monitoramento arqueológico) são empregados de acordo com as características e necessidades de cada projeto. Trata-se de uma prática adotada em todos os continentes, pelos países comprometidos com as normas de proteção ao patrimônio arqueológico preconizadas pela Organização das Nações Unidas para a Educação, a Ciência e a Cultura (Unesco), Conselho Internacional dos Monumentos e Sítios e Comitê Internacional de Gestão do Patrimônio Arqueológico.

Considerando ainda os demais bens acautelados, o Iphan também solicitou a avaliação de possíveis impactos ao patrimônio cultural imaterial registrados na área de influência do empreendimento, conforme o decreto n.3.551, de 4 de agosto de 2000, e a Convenção para a Salvaguarda do Patrimônio Cultural Imaterial, de 17 de outubro de 2003. Esta análise buscou identificar, portanto, as ocorrências de práticas de literatura de cordel, capoeira e do ofício das baianas de acarajé junto à comunidade local, conforme será apresentado, e possíveis interferências da obra.

## Breve histórico das pesquisas arqueológicas anteriores

O *campus* de Manguinhos, como qualquer outra ocupação humana de um determinado território, é proveniente de um processo histórico que resulta em paisagens socialmente construídas e gerenciadas, produto da interação das pessoas, do meio ambiente e das “coisas” em relação aos lugares (Anschuetz, Wilshusen, Scheick, 2001; Ashmore, 2004; Bender, 1993; Branton, 2009; Rotman, 2003; Tilley, 1994; Tilley, Cameron-Daum, 2017).

Das modestas instalações do Instituto Soroterápico Federal em 1900, construído na Fazenda de Manguinhos, passando pela construção do monumental conjunto arquitetônico histórico de Manguinhos nas duas primeiras décadas do século XX, e crescimento ao longo do século XX, o CMF conservou até a atualidade seu conjunto arquitetônico original, no qual se destaca o Castelo Mourisco (Benchimol, 1990; Oliveira, 2003).

Há indícios de que o local foi ocupado em tempos pré-coloniais por sociedades tupinambás, pelo menos há aproximadamente 1.500 anos, o que é atestado pela presença de fragmentos cerâmicos identificados no CMF na área do Laboratório do Pavilhão Rockefeller (Beltrão, 1978, 1989; Campos, 2014; Oliveira, 2002) (Figura 2).

Pesquisas realizadas ao longo dos últimos 20 anos em locais específicos do CMF – nas áreas do Pavilhão Lauro Travasso, do Parque da Ciência, do edifício da Hanseníase e seu estacionamento, do Centro de Documentação em História da Saúde e do Laboratório do Pavilhão Rockefeller (Figura 2) – geraram rico acervo de cultura material associado ao histórico de ocupação do local nos períodos colonial e republicano, compreendendo peças de faianças finas, porcelanas, cerâmicas, vidros, entre outras (Campos, 2014; Lima, 2011; Oliveira, 2002; Scaramella, 2017, 2019).

Por exemplo, nas áreas do Parque da Ciência e do Centro de Documentação em História da Saúde foram identificados – associados ao antigo Complexo de Incineração de Lixo Urbano do final do século XIX, descarte de lixo local gerado pela Fiocruz, e a aterros recentes – vestígios como plásticos, material vítreo e peças metálicas; assim como material laboratorial: ampolas, frascos de medicamentos e de produtos químicos, e instrumental de dutos e outras peças especiais feitas com o vidro bórico. Também foram coletados, em quantidades menores, fragmentos de faiança fina, porcelana, *stoneware*, metal e ossos de animais (Lima, 2011; Scaramella, 2017). O acervo arqueológico do *campus* de Manguinhos está separado por coleções, resultantes das pesquisas empreendidas, e encontra-se em três instituições de guarda: Museu Nacional (Lima, 2011), Laboratório de Arqueologia Brasileira da Universidade Federal do Rio de Janeiro (Campos, 2014) e Laboratório de Antropologia Biológica da Universidade do Estado do Rio de Janeiro (Scaramella, 2017).

Levando-se em consideração todos os estudos arqueológicos realizados no *campus* da Fiocruz (Figura 2), a área próxima ao Pavilhão Rockefeller/Parque da Ciência foi a que apresentou maior potencial arqueológico, sendo que as áreas no entorno do edifício da Hanseníase e seu estacionamento, e de expansão do *campus* de Manguinhos também apresentaram alto potencial arqueológico.

## Evidências arqueológicas

Durante 32 semanas de atividades relacionadas à implantação de tapumes, à execução de prospecções e mapeamento de interferências e às escavações de quatro poços do CT Faria-Timbó na área da Fiocruz foi realizado o monitoramento arqueológico. No início da execução das atividades no poço PV-245 constatou-se sua proximidade ao antigo muro do CMF, bem como questões logísticas que demandariam a abertura de uma passagem por esse muro; sendo assim, e no intuito manifestado pela Fiocruz de preservar esta estrutura, o PV-245 foi realocado (Figura 2).

**Figura 3:**
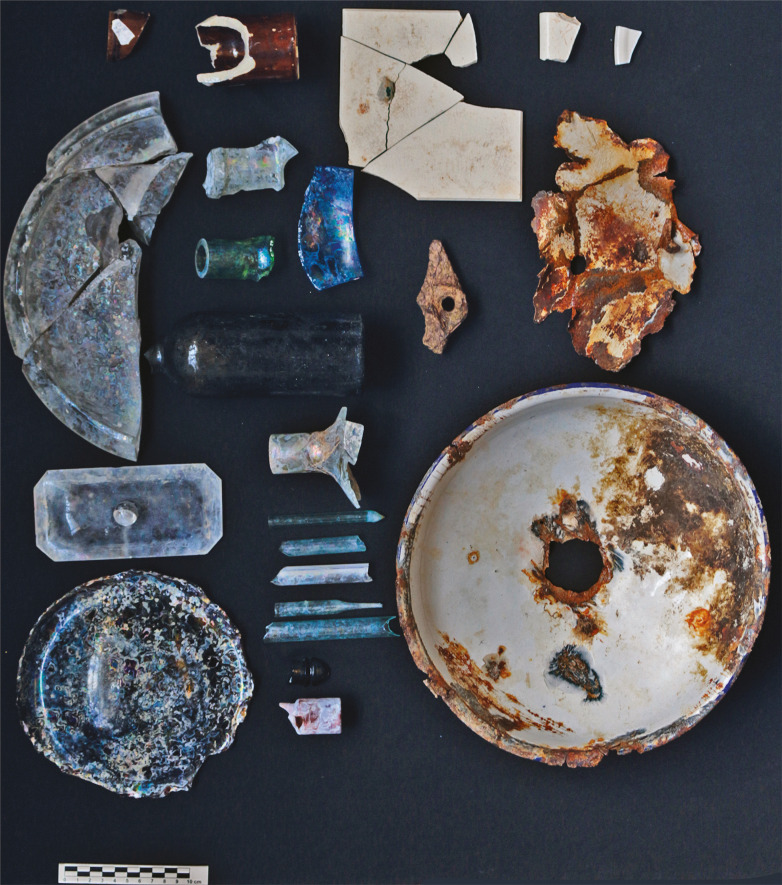
: Material identificado durante as escavações o poço PV-244 (foto: Renato Kipnis)

Ao longo das atividades iniciais da escavação dos poços PV-244 e PV-245 (Figura 2) foram identificados vestígios de cultura material histórica. Durante a escavação do poço PV-244, foram encontrados fragmentos arqueológicos em até 1,3m de profundidade. Materiais de uso cotidiano, como louça e garrafas (Figura 3), e material associado a atividades laboratoriais com vidro (Figura 3) e metal que remetem potencialmente às primeiras décadas de atividades da Fiocruz. Já na escavação do PV-245, a aproximadamente 1,5m de profundidade foi identificado um bolsão de entulho com material relativamente recente, como um pneu, restos de tijolos e de material de construção, e fragmentos de piso e de azulejos.

Nos dois poços de serviço, o material foi identificado em bolsões, “amontoados”, redepositados, sem nenhuma estruturação, em meio a sedimentos resultantes de aterro, transportados de área-fonte desconhecida. Ao todo foram exumados 81 artefatos de interesse, entre peças de barro (29), louça/porcelana (3), metal (5), vidro (39) e azulejo (5).

## Evidências imateriais

O processo de ocupação do território do *campus* de Manguinhos não ocorre separado de um contexto mais amplo, permeado por eventos traumáticos da nossa história nacional e mundial, como o processo de colonização, os conflitos com povos originários e a escravidão de pessoas retiradas do continente africano, os quais foram seguidos de outros movimentos de migração, disputa e transformação do espaço e de práticas culturais, resultando num palimpsesto que caracteriza o cenário de Manguinhos e seu entorno, manifesto material e imaterialmente.

A diáspora nordestina, movida pela fome e pela seca, dispersou um contingente de pessoas pelo território brasileiro, atuando diretamente nos processos de construção do país. Dirigindo-se inicialmente para o Norte, esses cidadãos foram chamados de “soldados da borracha”; posteriormente, deslocando-se para construção da nova capital federal, foram nomeados “candangos”; e, em outro período, espalhando-se para os estados de São Paulo e Rio de Janeiro, tornaram-se a principal mão de obra aplicada nos projetos desenvolvimentistas locais.

Felizmente, esses sujeitos trouxeram e conseguiram manter diversos elementos culturais, desde a música e a comida até as expressões, por meio de lugares de memória e resistência dessas manifestações culturais no Rio de Janeiro (Nemer, 2011).

Conforme mencionado, as exigências legais para o licenciamento ambiental e as leis de proteção ao patrimônio cultural indicam, também, a necessidade de pesquisas sobre os bens registrados como patrimônio cultural brasileiro na área de influência de empreendimentos. No caso do percurso da rede prevista para o CT Faria-Timbó, indicou-se que a avaliação de impacto deveria atentar para quatro bens registrados junto ao Iphan. Foram, portanto, considerados para este estudo as práticas da literatura de cordel, o ofício de mestres de capoeira, as rodas de capoeira e o ofício das baianas de acarajé.

A metodologia adotada para este estudo apoiou-se numa perspectiva etnográfica, baseada em entrevistas, mais especificamente, nos depoimentos dos sujeitos e agentes detentores dos saberes e práticas culturais, além da consulta a alguns especialistas e fontes bibliográficas, e uma avaliação de impacto ambiental. Todas as entrevistas foram gravadas, prezando assim por registros mais íntegros dos depoimentos, tanto para acompanhamento dos órgãos de proteção ao patrimônio quanto para formação de acervos que preservem a memória e possibilitem a realização de pesquisas futuras.^3^

**Figura 4:**
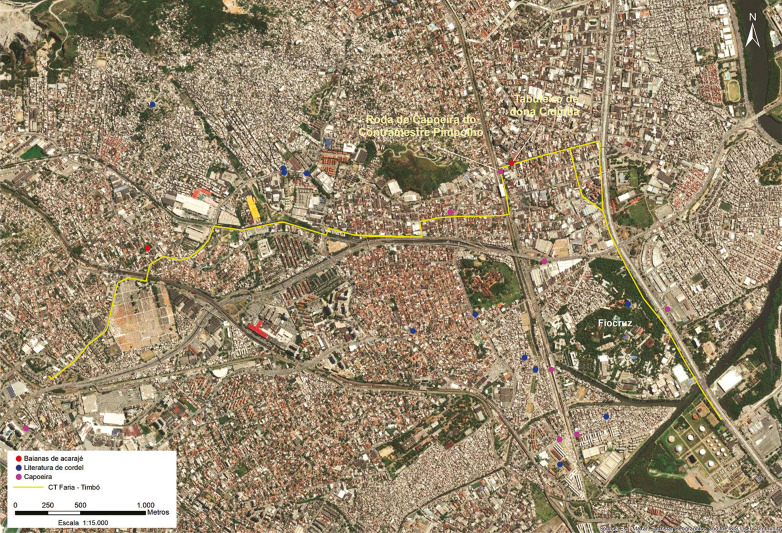
: Pontos de interesse para bens acautelados no entorno do Tronco Coletor Faria-Timbó-Fiocruz (GoogleEarth/Passarelli, autoria Renato Kipnis)

Importante destacar que, para tal estudo, se fazem necessários, além da identificação e localização georreferenciada das áreas de ocorrências dos bens, o mapeamento e a compreensão das relações entre as instituições de referência, os indivíduos e os lugares dessas práticas. Todos esses dados são cruzados com as informações do empreendimento e analisados em conjunto (Figura 4), para avaliação e prevenção ou proposições de mitigação e compensação de impactos.

## Literatura de cordel

Arte viva e ativa, a literatura de cordel foi registrada pelo Iphan em 2018, como uma forma de expressão, devido a sua importância em meio à diversidade de linguagens culturais do país. Movimento literário popular, múltiplo e amplo, o cordel tem berço no Nordeste brasileiro e, junto com o povo nordestino, espalhou-se pelo país, tornando-se parte do desenvolvimento educativo e comunicacional. Serviu para alfabetizar muitas crianças, algumas que, aliás, vieram a se tornar escritoras sem sequer acessar estudos formais. Cordelistas, juntamente com desenhistas, xilogravadores, cantantes, repentistas, comerciantes e tantos outros, narram e fazem circular histórias independentes; são “imprensa livre” (Helena citado em Iphan, 2018, p.8).

Cultura marginalizada, apesar de livre expressão do pensamento popular, as práticas relacionadas aos cordéis já foram muito desvalorizadas pelas elites intelectuais e até chegaram a ser criminalizadas no início do século XX (Iphan, 2018). No entanto, a literatura persistiu e se espalhou, criando pontos de resistência em vários lugares e, em especial, na cidade do Rio de Janeiro (Nascimento, 2019). Como medidas de salvaguarda, recomendam-se como ações importantes: a manutenção dos vínculos com a oralidade; a proteção do folheto impresso e de seus espaços tradicionais de difusão; a proteção ao direito autoral e a difusão do cordel na escola.

Dentre a área deste estudo, destaca-se a atuação do Coletivo Experimentalismo Brabo (Melo, Angelo, 2023), iniciativa que surgiu no Complexo de Favelas de Manguinhos propondo reflexões artísticas sobre “afeto, solidariedade e cultura de paz” e que produziu uma série de cordéis^4^ contando a história de diversos lugares e moradores do território.

Provocações do artista e cordelista Leo Salo, as narrativas homenageiam personagens que representam um movimento cultural específico, evidenciando a diversidade cultural ativa dessas comunidades, região que acabou ficando conhecida como “faixa de Gaza”, nomenclatura que destaca apenas a presença da violência no local. As obras difundem o gênero literário do cordel, destacando histórias como a do próprio Castelo da Fiocruz, referência local, do boteco da Lauzinha, ponto de encontro tradicional há mais de 20 anos na favela ou a de Iguinho Imperador, conhecido como o Rei na dança em Manguinhos. O projeto Memória de Manguinhos em Cordel produziu folhetos que são distribuídos gratuitamente em eventos incorporados à “estética da favela” e utilizados em oficinas de escrita dentro da comunidade.

José Franklin da Silveira, oriundo do Complexo do Alemão, escritor e cordelista, é um “carioca nordestino” que produz cultura de dentro da favela (Favrat, 2020). Chegou a comercializar cordéis na Feira de São Cristóvão e na Feira das Palmeiras, junto ao teleférico. Franklin liderou a iniciativa de montar uma “cordelteca”, com doações de vários cordelistas, espaço que, no entanto, se encontra fechado. Durante certo tempo, ele comercializou livros e cordéis numa banca na entrada da favela, mas, com a baixa das vendas, teve que fechar o ponto e passou a vender pela internet. Atualmente tem se adaptado aos novos recursos, produzindo conteúdo em um canal no Youtube.^5^ Um de seus cordéis de maior repercussão é *Dico e a invasão do Alemão*, que relata uma história paralela que ocorre no dia da “pacificação” do morro do Alemão.

Outro escritor e educador, Victor Alvim Itaim Garcia, conhecido como Victor Lobisomem, é um capoeirista e cordelista de Higienópolis. Um artista que tem em sua residência um acervo multicultural, contando com uma centena de cordéis, xilogravuras, além de álbuns, CDs e arquivos de pesquisa para as suas produções. Seu primeiro cordel foi feito para homenagear seu mestre de capoeira, mestre Camisa; essa iniciativa o levou a conhecer a Associação Brasileira de Literatura de Cordel e alguns dos cordelistas mais ativos no meio, como mestre Gonçalo Ferreira da Silva e José João dos Santos, o mestre Azulão. Integrando as duas referências, passou a realizar eventos promovendo a interação entre diversas práticas culturais, como orquestra de berimbau, roda de capoeira e a literatura de cordel. Hoje escreve, comercializa e desenvolve atividades educativas de forma autônoma,^6^ promovendo a poesia em festas literárias, rodas de samba e em escolas, com cordéis como o *ABC da capoeira para crianças*, de sua autoria.

## Capoeira, mestres e rodas

Atualmente reconhecida como prática cultural, a roda de capoeira está registrada como uma forma de expressão, e o ofício dos mestres de capoeira, como um dos saberes considerados patrimônio cultural imaterial do Brasil (Iphan, 2014). Originada no século XVII, após passar por um longo período de criminalização, a capoeira hoje é um dos maiores símbolos da identidade brasileira e está presente em todo o território nacional, além de ser praticada em mais de 160 países, em todos os continentes. No final de 2014, a roda de capoeira recebeu da Unesco o título de Patrimônio Cultural Imaterial da Humanidade.

O Rio de Janeiro foi um dos cenários de grande fluxo dessa prática. Na região de Manguinhos e Bonsucesso, a Turma dos Pimpolhinhos mantém rodas de capoeira permanentes, lideradas pelo contramestre Pimpolho, Alexandre Nascimento. O educador vivencia a capoeira desde os 6 anos, quando teve o primeiro contato por intermédio do Projeto Social Cemasi. Depois de adulto, o professor teve a oportunidade de reencontrar a mestra Cleide e o mestre Mintirinha, junto com o Grupo Terra, e seguir com sua formação. O desejo de dar início a um projeto próprio veio com a intenção de promover a capoeira dentro de Maguinhos, para comunidades como Mandela e Arara. O capoeirista é reconhecido no território por defender a inclusão na prática de crianças e idosos e pessoas LGBTQIA+, combatendo o preconceito.

## Baianas de acarajé

O registro do ofício das baianas de acarajé no Livro de Saberes do Iphan reconhece sua relevância para as tradições afro-brasileiras, que integram a cultura brasileira, sendo um importante símbolo de identidade étnica, regional e religiosa. Trata-se de um instrumento de reconhecimento oficial da riqueza e do enorme valor do legado de ancestrais africanos no processo histórico de formação de nossa sociedade (Iphan, 2007).

O dossiê apresenta uma série de características que formam um “sistema culinário”, amplamente conhecido como uma comida de rua, vendida por mulheres tipicamente vestidas como baianas e, eventualmente, também por homens. De acordo com Nina Bitar (2010, p.2), tal concepção “apreende a comida enquanto parte de um conjunto social e cultural, enfatizando as relações sociais e simbólicas em que ela está inserida e nas quais desencadeia efeitos”.

O acarajé, produzido incialmente no contexto do candomblé, como comida de santo, e posteriormente comercializado por mulheres, escravas ou libertas, que saíam pelas ruas com seus cestos ou tabuleiros na cabeça, relaciona-se intimamente a dois elementos importantes com relação às bases desse ofício no território: a rua, o “ponto”, e a construção de autonomia financeira feminina e familiar. São saberes construídos pelas mulheres negras trazidas para o Brasil, fruto de suas habilidades não só culinárias e empreendedoras, mas também daquelas que movimentaram e fortaleceram redes de resistência. É nas ruas que essas agentes estabelecem contatos, criam e reforçam laços e compõem a identidade cultural das cidades. Esses corpos (e vestimentas) ocupam papel fundamental no processo de patrimonialização e têm “como correspondência sua *performance* histórica pelas ruas da cidade, ou seja, a formação da identidade cultural da baiana se confunde com a modernização da cidade” (Rocha, 2007, p.76).

É importante destacar o papel da mulher negra e periférica, que está na base de nossa sociedade de estrutura ainda racista. No próprio Rio de Janeiro, “as tias baianas tomam conta do pedaço”, de acordo com Mônica Pimenta Velloso (1990, p.207); são essas mulheres que, mesmo em sua condição fragmentada, difusa, marginal e quase anônima, tratam incansavelmente de lutas do cotidiano, sabem de tudo um pouco, conhecem o poder das ervas, das rezas, dão conselhos, medeiam conflitos, administram recursos escassos, organizam festas, são importantes agentes comunitárias.

O consumo do acarajé, entretanto, não parece estar presente no cotidiano carioca; de acordo com os relatos, o alimento é mais procurado por turistas e por pessoas de origem nordestina, especialmente, aquelas vindas da Bahia. A pouca procura é avaliada pela questão do “custo-benefício” da comida, em relação às outras refeições, e sua comercialização tem sido mais rentável em áreas de maior poder aquisitivo, ou seja, na Zona Sul e no Centro da capital fluminense. Apenas dois pontos de comercialização foram identificados na área de estudo, os tabuleiros de “Dona Cidinha”, em Bonsucesso, e de “Dona Rita”, na Feirinha de Inhaúma, e a comercialização estava suspensa no período da pesquisa, que ocorreu em novembro de 2020, quando havia restrições sanitárias em virtude da pandemia de covid-19.

A Associação Nacional das Baianas de Acarajé e Mingau, Receptivos e Similares (Abam) tem atuado no Rio de Janeiro no intuito de organizar a distribuição geográfica dos “pontos” para instalação dos tabuleiros, além de fomentar a capacitação empreendedora das baianas filiadas. São oferecidos treinamentos com relação a cuidados sanitários e empreendedorismo. A atual coordenadora da Abam, Rosa Perdigão (2020), procura difundir e construir com as demais associadas a valorização do legado por elas herdado. Rosa reafirma o fato de que essas tias baianas não vendem só comida, também têm afeto, as pessoas vão em busca do axé.

## Considerações finais

Todo o material arqueológico identificado no presente estudo foi atribuído ao fim do século XIX e início do XX, similarmente ao material identificado nos estudos arqueológicos anteriormente realizados no *campus* da Fiocruz. É interessante notar que, apesar de o material não apresentar nenhuma estruturação e ocorrer em área não identificada anteriormente como contendo alto potencial arqueológico, as categorias de cultura material recuperadas abrangem grande variedade.

Não foi identificado nenhum vestígio que remetesse a uma ocupação pré-colonial. Também não foram identificados piso ou estrutura que indicassem a presença de edificação nos locais monitorados.

A história da ocupação do atual *campus* da Fiocruz remonta ao período pré-colonial, com a presença de povos falantes da língua tupi-guarani que ali constituíram suas aldeias, há aproximadamente 1.500 anos, e permanecem até os dias atuais. Com a chegada dos colonizadores europeus a área começa a sofrer mudanças, com a instalação do Engenho da Pedra, ao final do século XVIII, depois da Fazenda de Manguinhos, na primeira metade do século XIX, e, posteriormente, no final do século XIX, com a implantação de fornos destinados à cremação do lixo urbano da cidade e do Instituto Soroterápico Federal. Vestígios arqueológicos desse processo histórico estão presentes e preservados de diferentes modos e contexto no *campus* da Fiocruz, o que demonstra e justifica a necessidade de pesquisas arqueológicas e medidas mitigadoras para proteção desse valioso patrimônio.

As entrevistas com os detentores versaram sobre as histórias e trajetórias de vida, buscaram compreender as relações dos sujeitos com o território e com o bairro, especialmente para aqueles localizados mais próximos à área diretamente afetada pelas obras de instalação da rede de esgotos, além de buscar elementos para compreensão das relações com suas referências culturais, especificamente com as características de expressividade no contexto do Rio de Janeiro.

A avaliação de impacto se concentrou na análise de dois pontos de ocorrência muito próximos do empreendimento (Figura 4), o “ponto” do tabuleiro de dona Cidinha, e a roda de capoeira do contramestre Pimpolho, ocorrências desenvolvidas na praça das Nações, ao lado da estação Bonsucesso. Em diálogo com os detentores, buscou-se compreender se haveria a possibilidade de as atividades da obra causarem alguma interferência negativa em suas práticas.

Com relação à comercialização do acarajé, as atividades já estavam sofrendo o impacto das restrições impostas pela pandemia. Dona Cidinha teve que optar por não montar o tabuleiro alguns dias, pois acabava tendo prejuízos com produção não comercializada, mas tinha receio de perder seu ponto junto aos demais comerciantes da praça. O ponto do tabuleiro fica entre duas áreas de intervenção do empreendimento: PV-235 (100m) e PV-236 (425m). Durante a entrevista, dona Cidinha relatou já ter percebido a obra se instalando, apesar de não saber do que se tratava nem constatar interferência na sua comercialização até o momento.

Com relação à prática de capoeira no local, identificou-se que a tradição do grupo era de realizar a roda aos sábados à noite, em um local a apenas 25m de um dos pontos de perfuração da obra (PV-235). Como as intervenções no espaço necessitam de sinalização e certo isolamento de segurança no entorno, avaliou-se a possibilidade de prejuízo para a prática, como, por exemplo, restrições de acesso ou interferência sonora excessiva. Em consulta aos próprios trabalhadores da obra constatou-se que as atividades, aos sábados, encerravam-se até no máximo as 17h; dessa forma, o horário de atividades do empreendimento não coincidiria com a prática de capoeira no local. As rodas também estiveram suspensas, considerando as orientações de distanciamento social, mas preparavam-se para retomar as atividades presenciais. Para ambos os casos, os responsáveis pelo empreendimento foram alertados da proximidade com os pontos de interesse, para que tomassem todas as medidas necessárias à prevenção de qualquer impacto.

## NOTAS

1 A pesquisa de campo relacionada ao patrimônio imaterial ocorreu em novembro de 2020, sob a responsabilidade de Carlos E. Caldarelli, Ilza Carla F. de Lima e Marcelo M. de Medeiros. O projeto de pesquisa arqueológica (i.e., monitoramento arqueológico) ocorreu entre janeiro e agosto de 2021 com coordenação geral de Renato Kipnis e tendo como coordenadora de campo a arqueóloga Flávia Costa. O projeto foi aprovado pelo Iphan, por meio da portaria n.79 de 30 de dezembro de 2020.

2 Território como unidade espacial resultado do uso cumulativo da terra e seus recursos e das interações humanas em todos os seus aspectos socioeconômico-culturais ao longo do tempo (Zedeño, 1997).

3 Os interessados em pesquisar o acervo de audiovisuais produzidos com esta pesquisa podem entrar em contato com os autores.

4 A coleção Memória de Manguinhos em Cordel está disponível em: https://ebrabo.wordpress.com/projetos/cordel-favela-historia-memoria/.

5 Os cordéis de José Franklin podem ser acessados em seu canal no Youtube: https://www.youtube.com/channel/UCg3XeWIMl_eXfwConDnRQ6A/featured.

6 O cordelista Victor Lobisomem mantém divulgação e comercialização da sua arte nas redes sociais, como o Instagram: https://www.instagram.com/victorlobisomem/.
